# The anterior-ventrolateral temporal lobe contributes to boosting visual working memory capacity for items carrying semantic information

**DOI:** 10.1016/j.neuroimage.2017.12.085

**Published:** 2018-04-01

**Authors:** Rocco Chiou, Matthew A. Lambon Ralph

**Affiliations:** The Neuroscience and Aphasia Research Unit (NARU), Division of Neuroscience and Experimental Psychology, School of Biological Sciences, University of Manchester, UK

## Abstract

Working memory (WM) is a buffer that temporarily maintains information, be it visual or auditory, in an active state, caching its contents for online rehearsal or manipulation. How the brain enables long-term semantic knowledge to affect the WM buffer is a theoretically significant issue awaiting further investigation. In the present study, we capitalise on the knowledge about famous individuals as a ‘test-case’ to study how it impinges upon WM capacity for human faces and its neural substrate. Using continuous theta-burst transcranial stimulation combined with a psychophysical task probing WM storage for varying contents, we provide compelling evidence that (1) faces (regardless of familiarity) continued to accrue in the WM buffer with longer encoding time, whereas for meaningless stimuli (colour shades) there was little increment; (2) the rate of WM accrual was significantly more efficient for famous faces, compared to unknown faces; (3) the right anterior-ventrolateral temporal lobe (ATL) causally mediated this superior WM storage for famous faces. Specifically, disrupting the ATL (a region tuned to semantic knowledge including person identity) selectively hinders WM accrual for celebrity faces while leaving the accrual for unfamiliar faces intact. Further, this ‘semantically-accelerated’ storage is impervious to disruption of the right middle frontal gyrus and vertex, supporting the specific and causative contribution of the right ATL. Our finding advances the understanding of the neural architecture of WM, demonstrating that it depends on interaction with long-term semantic knowledge underpinned by the ATL, which causally expands the WM buffer when visual content carries semantic information.

## Introduction

Working memory (WM) is a vital cognitive faculty that holds information in a temporary cache. Information kept in WM is used to guide cognition (e.g., whilst reading, bearing words early in a sentence in mind to apprehend the whole) and action (e.g., whilst parking a car, remembering the completed steps of manoeuvre before performing the next). In the domain of visual WM, most researchers use abstract stimuli, such as colour patches, in an attempt to gauge the bare capacity while insulating it from the ‘contamination’ of existing knowledge (for review, see Brady et al., 2011). However, this practise of using abstract stimuli draws criticisms that it lacks ecological validity and underestimates the real capacity of WM (Wyble et al., 2016). Whilst there is considerable convergent evidence from patients and healthy participants that long-term semantic knowledge benefits verbal WM (e.g., Jefferies et al., 2006a, Jefferies et al., 2006b), it remains somewhat equivocal whether it has analogous effects on visual WM. In a behavioural study, Curby et al. (2009) found that expertise in cars enables experts to remember more cars than do novices. In a recent study, Brady et al. (2016) explored how electrophysiological response varies when the WM system is storing objects *v*s. colour patches. They looked specifically at the contralateral delay activity (CDA), a neural signature that diminishes in amplitude when information transits from WM to episodic memory. Results showed that WM capacity for objects increased as a function of longer encoding time, which was reflected in significantly greater CDA. By contrast, WM capacity for colours remains constant despite more encoding time, which was mirrored in a smaller magnitude of CDA. These indicate that, compared to meaningless abstract stimuli, entities that hold ecological relevance (e.g., objects encountered daily in the visual environment) are tackled differently by the WM system, presumably aided by semantic knowledge or familiarity with objects' configuration.

Advancing from the electrophysiological correlates discovered by Brady et al., in the present study we investigated the causative neural regions and mechanisms that mediate this boosting impact of ecologically relevant stimuli on WM capacity. We focused on the obvious yet untested hypothesis that the effect hinges on semantic knowledge (the long-term representation for the meaning of objects, people, words, etc.). We utilised the ecologically salient test-case of human faces and compared faces that are highly recognisable and carry semantic information (celebrity faces) *vs*. faces without prior exposure (unfamiliar faces). Colour patches were included as control stimuli. We selected faces as the target stimuli based on the following considerations: First, faces are of paramount ecological importance, crucial for survival, which would be endowed with expanded WM storage to fulfil the need of social interaction. Second, by comparing celebrity and unfamiliar faces, we are able to glean insight into how person-related semantic knowledge affects WM capacity. Third, face processing is associated with a well-defined neural network, which enables us to target a specific neural locus, transiently unsettle its functioning using non-invasive neurostimulation, and test the interactive impacts of brain stimulation and experimental factors.

It is well-established that face processing is supported by a neural network distributed along the ventral occipitotemporal cortex, starting from the occipital face areas (OFA) sensitive to componential face features, through the fusiform face areas (FFA) sensitive to holistic configuration of face-like stimuli, and culminating in the anterior temporal lobe (ATL). At the apex of this hierarchy, the ATL codes face identity and person-related attributes (Collins and Olson, 2014), as well as semantic knowledge about other domains more generally (e.g., objects, words, etc.; Lambon Ralph et al., 2017). There has been plenty of evidence that the ATL houses a face-sensitive area that is tuned to person identity while unaffected by changes of facial expression (Nestor et al., 2011) and viewpoint (Anzellotti et al., 2013). Amongst the face-sensitive regions of the occipital and temporal cortices, the right ATL is the only area exhibiting adaptation (decline in activity) to repeated exposure to the same person's different faces (Yang et al., 2016) and is able to integrate the neural coding of face and headless body to represent a whole person (Harry et al., 2016). Moreover, multivariate pattern analysis has shown that the ATL contains information allowing cross-classification between faces and names (Wang et al., 2017). Based on abundant evidence, we targeted the ATL to investigate whether this neural structure causally underpins any potential impact of person identity (and by extension semantic knowledge more generally) on WM storage.

Due to its adjacency to facial nerves, stimulating the ATL evokes twitches of facial muscles. To ascertain that effects of ATL stimulation cannot be driven by non-specific factors (e.g., twitches or discomfort), in addition to the conventional control site of the vertex we included the lateral prefrontal cortex (PFC) as a control site. The lateral PFC serves as a suitable control region because it is also near facial nerves (hence eliciting similar twitches during stimulation). Besides serving as a control site, the inclusion of PFC allowed evaluating a region that underpins executive processes but does not represent the content of WM and semantic knowledge (Lambon Ralph et al., 2017, Postle, 2016). Specifically, in the WM literature, different subdivisions of the PFC have been found to strengthen its connectivity with the FFA when faces are being maintained in WM (Bollinger et al., 2010, Chadick and Gazzaley, 2011, Lee and D'Esposito, 2012). This has been interpreted as the PFC generating modulatory signals that fortify the actual content of WM (representations of faces) stored in the FFA, expediting the processing of relevant stimuli (faces) while curbing further processing of distracting stimuli (Gazzaley and Nobre, 2012). Thus, PFC stimulation mimics tactile sensation that ATL stimulation produces and allows testing whether an executive-related region contributes to a semantically-mediated effect.

We report two experiments in this study. Experiment 1 was a psychophysical investigation in which we independently manipulated different types of stimuli and encoding duration. We established a robust effects that faces were endowed with an advantage of WM storage over abstract stimuli, and this advantage is further enhanced for faces associated with person-related knowledge. Experiment 2 was a neurostimulation investigation in which we exploited theta-burst stimulation to impede the processing of the ATL, while the PFC and the vertex were included as control sites to ensure the specificity of ATL stimulation.

## Materials & methods

### Participants

Twenty-seven volunteers (Experiment 1: n = 15, eight females, mean age: 26 ± 5; Experiment 2: n = 12, seven females, age: 29 ± 8), gave informed consent. All participants are right-handed, had normal vision, completed safety screening for TMS and MRI before the experiments, and reported no history of neurological or psychiatric issues. This study was reviewed and approved by the local research ethics committee.

### Apparatus

For the transcranial magnetic stimulation (TMS) experiments, we first acquired a high-resolution T1-weighted anatomical image for each volunteer using a 3T Philips Achieva scanner equipped with an 8-element head-array coil; in-plane resolution was 0.94 mm; slice thickness was 0.9 mm. In the subsequent sessions, we conducted behavioural testing combined with TMS. Visual stimuli were shown using MATLAB with Psychtoolbox (Brainard, 1997, Pelli, 1997) on a computer monitor (29 × 39.5 cm; 75 Hz refresh rate; 1024 × 768 resolution). Head position was stabilised with a chin-rest, keeping a viewing distance of approximately 57 cm from the screen. Brain stimulation was applied via a Magstim Super Rapid^2^ system equipped with a figure-of-eight coil (70 mm). Stimulation was guided using Brainsight *2* (Rogue Research Inc.), a frameless stereotaxic neuronavigation system that allows precise targeting and flexible calibration (see below for detailed protocols).

### Experiment 1

**Fig. 1:**
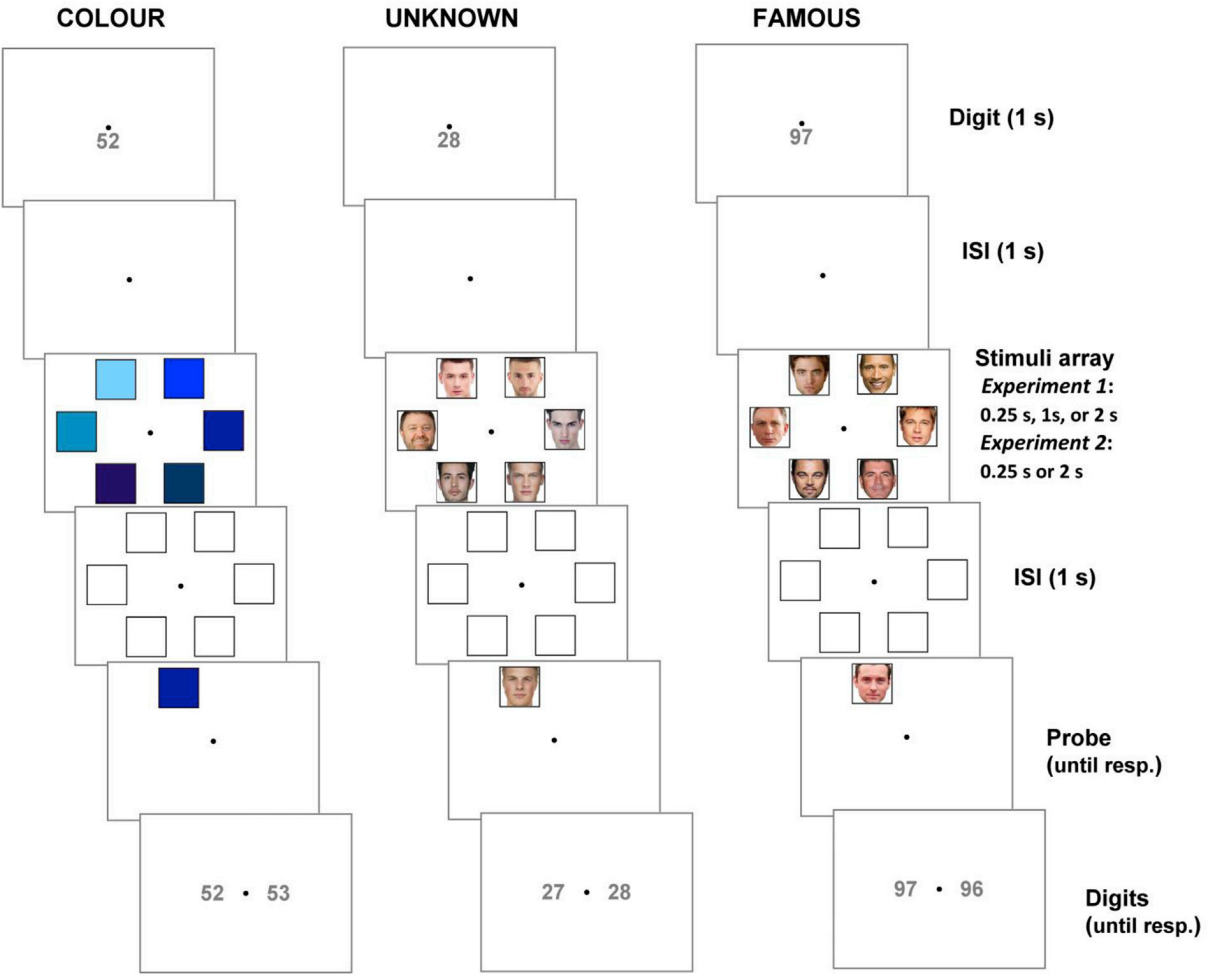
Timeline of events occurred during a trial. Participants viewed coloured shades, unknown faces, or famous faces for differential amount of time. They were asked to respond to the visual WM task before the verbal interference task (rehearsing a two-digit numeral).

Experiment 1 was a psychophysical investigation with twofold aims to test (*i*) whether WM capacity would be boosted for human faces, compared to abstract stimuli and (*ii*) whether WM would be further augmented for famous faces, compared to unknown ones. We used a 3 × 3 within-participant factorial design, with Stimuli (colour patches, unknown faces, and famous faces) and Encoding Duration (0.25 s, 1 s, and 2 s) as repeated-measure variables. Built upon a similar design of Brady et al. (2016), we required participants to perform a visual working memory task (see Fig. 1). At the beginning of each trial, we presented a two-digit numeral, situated below the central fixation dot. Participants were told to covertly rehearse the number throughout the trial. After an inter-stimulus-interval (ISI, 1 s), they were presented with a circular array consisting of six visual items (shown inside placeholders) evenly spaced along an invisible circumference (16.5° diameter). In different conditions, the stimuli were six coloured squares, six unknown faces, or six famous faces, appearing for 0.25 s, 1 s, or 2 s, followed by a 1-sec ISI during which the placeholders remained visible while all stimuli inside had disappeared. Subsequently, participants were tested with a probing item, randomly appeared in one of the six locations from the initial array. The probe remained on the screen until participants indicated with a button response whether the stimulus was identical to (50% of the trials) or different from (50%) the item that appeared at that particular location in the original array. After a response was made, the probe was cleared from the screen and replaced with two numeral options; one two-digit number situated on the left and the other one the right. Participants had to indicate which one was the number that they had been rehearsing during the trial. The numbers remained visible until a response was made, which was then followed by a 1-sec inter-trial-interval. We used the identical structure of design to Brady et al. (2016) in that each condition (3 Encoding time × 3 Stimuli) was perform in a single block of 40 trials, with the order of the nine blocks randomised across participants. The experiment contained 360 trials in total and took approximately 60 min.

One hundred and twenty well-known, highly recognisable faces (sized 6.5° × 6.5°) were used for the ‘famous’ condition. These images contained 60 male faces and 60 female faces. The faces included politicians, athletes, and celebrities widely known in the United Kingdom. These 120 faces were selected from a much larger set of faces, unanimously judged by five UK residents as highly familiar. Another 120 faces (sized 6.5° × 6.5°, 60 males, and 60 females) were used in the ‘unknown’ condition; these faces were also selected from a larger set of materials, all judged as unfamiliar. Faces and locations were randomly permutated to ensure each array contained a novel combination of constituents. To prevent strategic grouping within an array, male and female faces were presented in separate trials.

It is noteworthy that Brady et al. (2016) presented objects of saliently distinct colours and shapes within an array (e.g., blue bucket, yellow brush, etc.). By contrast, human faces can only vary in a limited range of complexion colours (from light to dark skin). To match the variation in the face conditions, in the colour condition we used six different shades of blue/green squares (6.5° × 6.5°) within an array. While the six shades shared the same generic colour label, they were readily distinguishable from each other (see Table 1 for RGB values and illustrations); the blue (50%) and green arrays (50%) were displayed in separate trials.

**Table 1:**
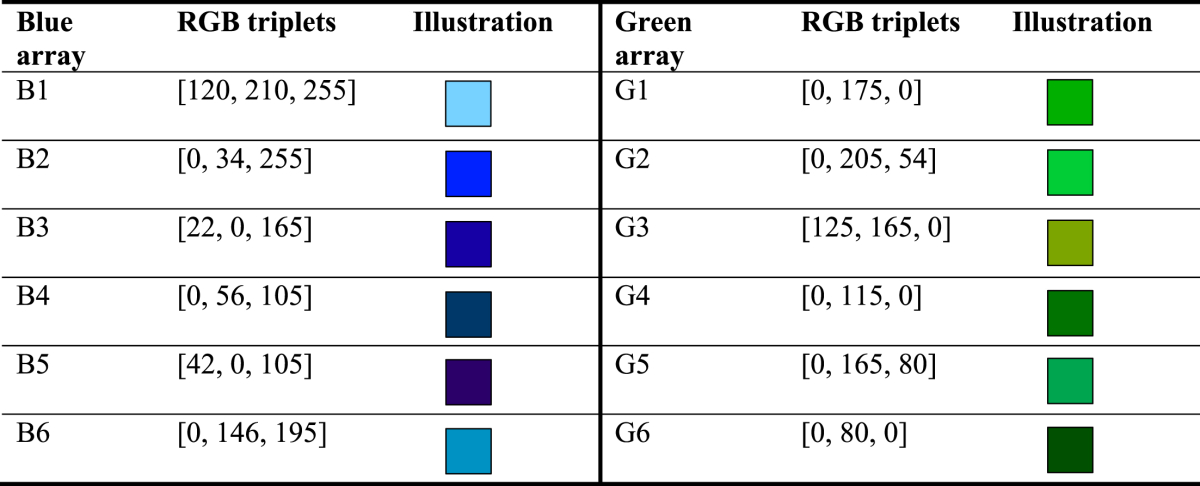
RGB values of the six colours displayed in a blue/green array, and their illustrations. Each colour appeared randomly in one of the six locations of an array, varying from trial to trial.

Of particular concern is whether the famous and unknown faces are matched for low-level visual statistics. It has been demonstrated that WM performance tends to improve when the items are visually distinct from each other, relative to them being less distinguishable (Jiang et al., 2000). Thus, it is necessary to ascertain the degree to which the famous male/female faces are differentiable from each other has been equated to that of the unknown male/female faces. To verify equivalent visual distinctness between the two face conditions, we computed and compared their low-level visual properties using the GIST descriptor algorithm (Oliva and Torralba, 2001). This algorithm has been extensively applied to compare visual similarity between images, including faces (Rice et al., 2014) and objects (Chiou and Lambon Ralph, 2016). For each individual image, we first passed it through a series of Gabor filters across eight orientations and four spatial frequencies, giving 32 filtered images. These were subsequently rendered along a 4 × 4 grid to derive a GIST descriptor (a vector of 512 values), which characterised an image in terms of its spatial frequencies and orientations present at different locations scattered across the image. In the final step of this analysis pipeline, we computed *pair-wise* visual distance by comparing their GIST descriptors, iterated the computation for the exhaustive combination of 1770 pairs, and performed separately for famous/unknown faces of males/females (note that faces of different sexes and familiarity were shown in separate trials). The resultant measure of visual similarity (1 minus GIST distance) ranged from zero to one, with a higher value indicating greater visual similarity. Comparisons of image similarity between conditions showed that the extent of similarity between famous male/female faces has been matched to that of unknown male/female faces, supported by statistics (mean ± SD – famous males: 0.798 ± 0.073, unknown males: 0.799 ± 0.080, *p* = .68, *n.s*.; famous females: 0.774 ± 0.067, unknown females: 0.771 ± 0.078, *p* = .22, *n.s*.). In addition to controlling for GIST, we also ensured that luminance is matched between the conditions – we averaged the pixel-wise values of luminance for each image and compared between famous and unknown faces. Results revealed no significant difference between the two conditions (*t*_(238)_ < 1, *p* = .65, *n.s.*). Together, these careful procedures ensured that the famous and unknown faces have been well-matched on low-level visual characteristics. Thus, any difference of WM performance between the face conditions cannot be attributed to one set of stimuli being more distinguishable than the other set.

### Experiment 2

Experiment 2 was a TMS experiment to investigate the neural structures that causatively underlie the functional impact of face identity on WM capacity. We selected the ATL as the target, while the PFC and vertex served as control sites. Their neuroanatomical definition was based on relevant fMRI studies about the neural correlates of face perception and visual working memory. For the ATL, stimulation was guided using the peak locus of a face-sensitive cluster situated in the right anterior fusiform gyrus, based on the Rossion et al. (2012) study in which they compared normal faces against phase-scrambled faces and cars, and tested a sizeable sample of participants, (n = 40, coordinates: [28, 0, −28] in the Talairach space). For PFC stimulation, we selected a point located in the middle frontal gyrus (MFG, coordinates: [40, 32, 24] in the MNI space), identified by the Nee et al. (2013) meta-analysis. This meta-analysis surveyed 36 fMRI studies and found this right MFG site to be robustly engaged by WM processing of visual objects and spatial positions. The conventional control site of the vertex was defined using anatomical landmarks, as the midpoint between each individual's nasion and inion, along the sagittal midline of the scalp.

We used a 3 × 3 × 2 within-participant factorial design, with TMS target (ATL, MFG, and vertex), Stimuli (colours, unknown faces, and famous faces) and Encoding Duration (0.25 s, 2 s) as repeated-measure variables. The psychophysical procedure and stimuli were identical to Experiment 1, except the following modifications: instead of presenting trials of different encoding duration in separate blocks like our Experiment 1 and Brady et al. (2016), here the trials of 0.25-sec and 2-sec were randomly mingled within a block of 80 trials while the three types of stimuli were shown in separate blocks. In each TMS session, participants completed three blocks of 80 trials, giving 40 trials in each condition. The order of different stimuli and TMS targets were fully counterbalanced across participants.

For brain stimulation, we adopted offline continuous theta-burst stimulation (cTBS). Stimulation was delivered onto the targeted site in repeated trains of 300 bursts (3 magnetic pulses per burst; 50 Hz) with an inter-train-interval of 200 ms (5 Hz); the stimulation lasted for 60 s, with a total number of 900 magnetic pulses (Huang et al., 2005). Participants received cTBS prior to performing tasks; we probed their performance *immediately* following stimulation. This offline cTBS approach avoids non-specific interferences such as discomfort, noise, muscle twitches and so on, that online TMS (i.e., concomitant stimulation during task execution) usually produces and is suggested to be effective for probing high-level cognitive functions (Sandrini et al., 2012). The stimulation was set at 80% of resting motor threshold (RMT, the minimum stimulation intensity on the motor cortex that caused a visible finger twitch; for testing individual RMT, we applied single-pulse stimulation to the right primary motor cortex; the value was defined as the minimum strength sufficing to trigger visible twitches in the left abductor pollicis muscle on six out of ten contiguous trials). The averaged intensity of stimulation was 44 ± 1% of the stimulator maximum output (range: 42%–45%).

**Fig. 2:**
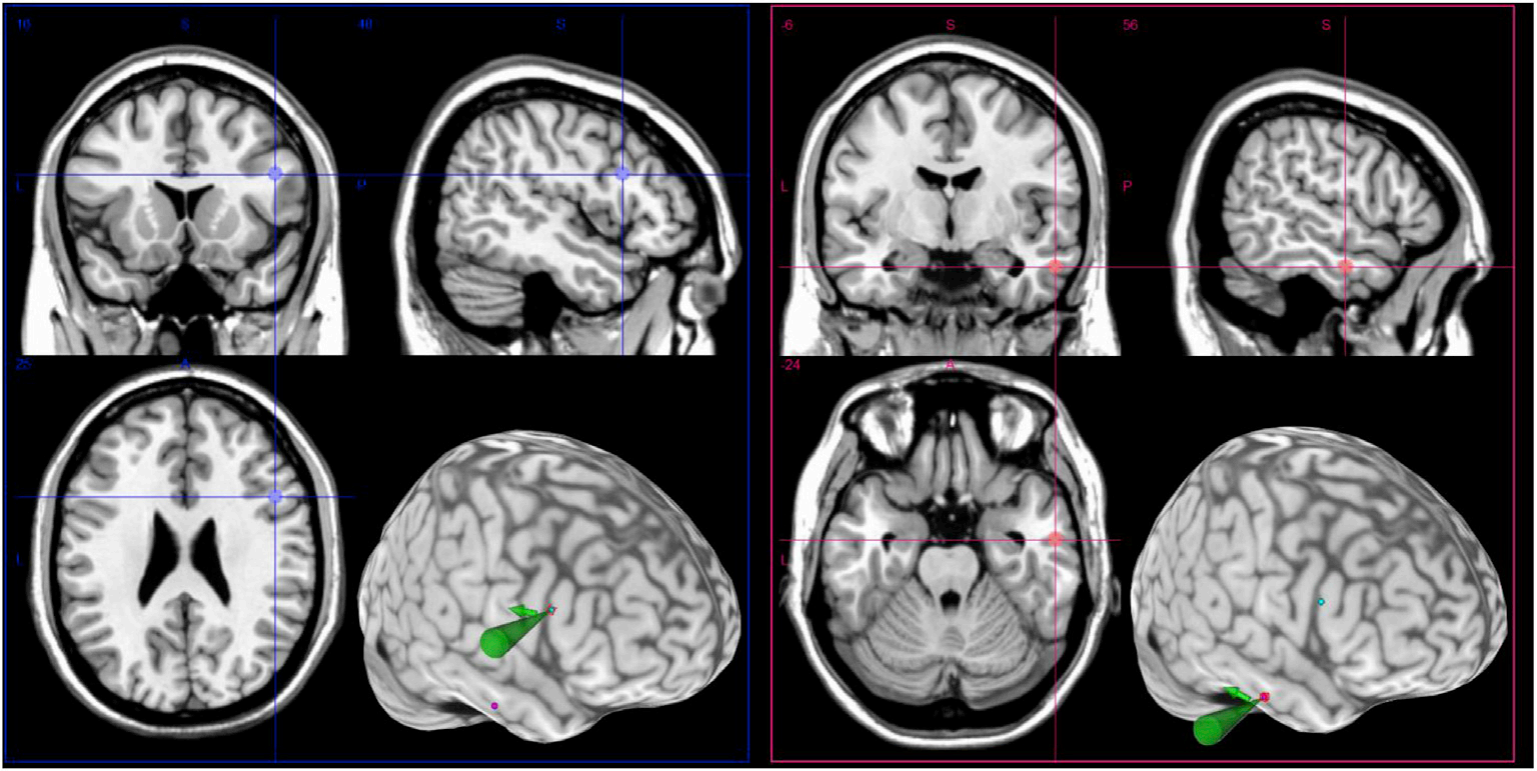
Target stimulation sites pinpointed on the MNI cortical template. Blue represents the right MFG. Red represents the right ATL.

Target sites for cTBS were localised individually based on T1-weighted structural image and co-registration between cortex and scalp. For each participant, we normalised the T1 image into the standardised space of MNI system using the ‘Segment’ function of SPM8 (Wellcome Department of Imaging Neuroscience, UK), and converted the coordinates of the literature-defined ATL and MFG sites to derive the corresponding coordinates in each participant's anatomical native space. As the location of the ATL site is slightly too ventral and medial to be accessed by stimulation on the scalp, we adjusted the location of this stimulation target based on individual anatomy, making it more lateral and dorsal to the original site and hence accessible to TMS. Meticulous care was taken to strike a balance between adjacency to the original converted site and accessibility to the more lateral scalp stimulation point. The MNI coordinates of the ATL and MFG sites averaged across participants were [56 ± 3, −6 ± 4, −24 ± 3] and [48 ± 3, 16 ± 4, 25 ± 3], respectively (see Fig. 2). Note that while the adjusted locus of ATL stimulation is lateral to the peak coordinate of Rossion et al. (2012), it still concords closely with prior literature of face recognition and personal-related knowledge. Multiple studies have reported robust activation of the right ATL, encompassing both the ventromedial and ventrolateral subregions. For example, Von Der Heide et al., (2013) used famous faces and reported three ATL loci ([25, 6, −24]; [45, 4, −26]; [54, −10, −24] in Talairach). In particular, the locations of their two lateral loci correspond closely with our right-ATL target. Furthermore, Wang et al. (2017) used MVPA and showed that various loci of the right *lateral* ATL contains information that allow cross-classification between faces and person-related attributes, which also overlap with our right-ATL target. Intriguingly, our ATL target falls in close proximity to the centre-point of hypo-metabolism ([54, 9,-24] in MNI; Zahn et al., 2009) in patients showing deficits about person-related knowledge. Taken together, multiple threads of evidence have indicated that the actual cortical regions that we stimulated – the right lateral ATL – is involved in some crucial aspects of face-processing, particularly in relating faces to individual identities and personality traits.

Before the WM experiments, we performed a co-registration procedure mapping the cerebral site of TMS target of each session onto the corresponding point on the scalpal surface using the Brainsight *2* neuronavigation system, which tracked the position of the coil during stimulation and allowed online adjustment to achieve accurate positioning. For all three stimulation sites, the coil was placed tangentially to the scalp with the handle pointing posteriorly (parallel to the rostral-caudal axis). For each individual, the TMS sessions were separated by at least 48 h.

## Results

### Experiment 1

**Fig. 3:**
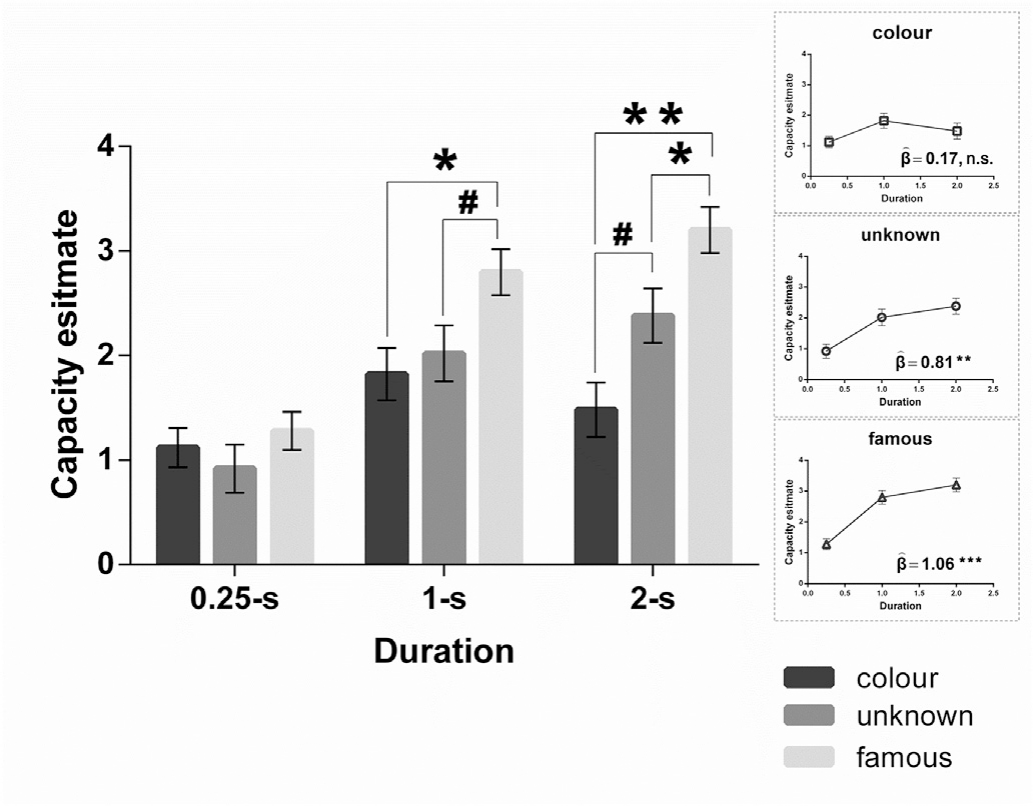
Capacity estimates (Cowan's *K*), representing the number of correctly remembered items, displayed as a function of the types of stimuli by encoding durations. * represents 0.05 > *p* > .01; ** represents 0.01 > *p* > .001; *** represents *p* < .001; # represents marginal significance (0.1 > *p* > .05).

Participants performed well on the digit task, achieving overall accuracy of 89% (SD: 3%). For each condition, we computed its estimate of WM capacity using Cowan's *K* proposed by Cowan (2001), formulated as *K* = SetSize × [(Hit Rate) – (False Alarm Rate)], indicating the number of accurately remembered items maintained in WM. The capacity estimates of all experimental conditions are displayed in Fig. 3. In the colour condition, we found that the duration of 0.25 s allowed participants to encode and sustain approximately one colour square. Crucially, we replicated previous findings (e.g., Bays et al., 2011, Brady et al., 2016, Luck and Vogel, 1997) that little to virtually no additional information about colour squares was accrued over the remaining 1750 msecs, such that the slope from 0.25 s to 2 s was not significantly different from zero (*β* = 0.176, *t*_(14)_ = 0.87, *p* = .39, *n.s*.; the upper inset box of Fig. 3). By marked contrast, in the other two face conditions, the number of faces remembered continued to accumulate from 0.25 s to 2 s, as supported by the significantly positive slopes in the conditions of unknown faces (*β* = 0.809, *t*_(14)_ = 4.33, *p* = .001; the middle box) and celebrity faces (*β* = 1.059, *t*_(14)_ = 5.56, *p* = .00007; the lower box). As evident in the bar graph of Fig. 3, there were significant main effects of Stimuli (*F*_(2,28)_ = 8.47, *p* = .001, *η*_*p*_^2^ = 0.38) and Encoding Duration (*F*_(2,28)_ = 33.89, *p* < .001, *η*_*p*_^2^ = 0.71). Crucially, the Stimuli × Duration interaction was statistically significant (*F*_(4,56)_ = 2.73, *p* = .03, *η*_*p*_^2^ = 0.16). Based on the significant interaction, we examined simple main effects by different durations and conducted *a posteriori* comparisons where appropriate. At the briefest 0.25-sec duration, the simple effect of Stimuli was not significant (*F*_(2,28)_ < 1, *p* = .51, *η*_*p*_^2^ = 0.04, *n.s*.), indicating that WM capacity for the three types of contents did not differ from one another. At the intermediate 1-sec duration, however, there was a significant effect of Stimuli (*F*_(2,28)_ = 3.37, *p* = .04, *η*_*p*_^2^ = 0.19). *Post-hoc* tests revealed that WM capacity for famous faces significantly surpassed that for colours (*p* = .008) and showed a trend greater than unknown faces (*p* = .08). At the longest 2-sec duration, the simple effect of Stimuli also reached significance (*F*_(2,28)_ = 9.56, *p* = .001, *η*_*p*_^2^ = 0.4). *Post-hoc* tests indicated that, under this lengthy duration, the advantage for famous faces persisted, making its storage significantly exceeded both colours (*p* = .001) and unknown faces (*p* = .02); moreover, the superiority of unknown faces started to emerge, making it marginally greater than colour patches (*p* = .059).

It has been suggested that the number of items held in WM and the precision/fidelity of WM content quantify distinct dimensions of memory ability (e.g., Awh et al., 2007). This claim has led to follow-up investigations into whether WM capacity is constrained by a limited set of items (how many) or limited amount of resources (how precise) (for discussion, see Brady and Alvarez, 2015). Cowan's *K* is a measure that taps into the number of WM items, which might not be suitable for the colour condition where there was no conspicuous variation between hues. Thus, we calculated performance accuracy (proportion of correct responses), which might better characterise WM when participants were limited by precision (e.g., difference between two hues). The accuracy results are entirely consistent with the *K* measures: For colour squares, the slope as a function of Encoding Duration was not different from zero (*β* = 0.009, *t*_(14)_ = 0.67, *p* = .52), indicating little or no improvement. By contrast, for unknown and famous faces, accuracy significantly increased with longer viewing time (unknown: *β* = 0.048, *t*_(14)_ = 3.18, *p* = .007; famous: *β* = 0.081, *t*_(14)_ = 4.38, *p* = .001). Taken together, these results suggest that (*i*) the WM system has greater capacity for retaining human faces relative to meaningless stimuli and (*ii*) faces that carry abundant identity-level information are kept better in WM than unfamiliar faces devoid of semantic information.

### Experiment 2

**Fig. 4:**
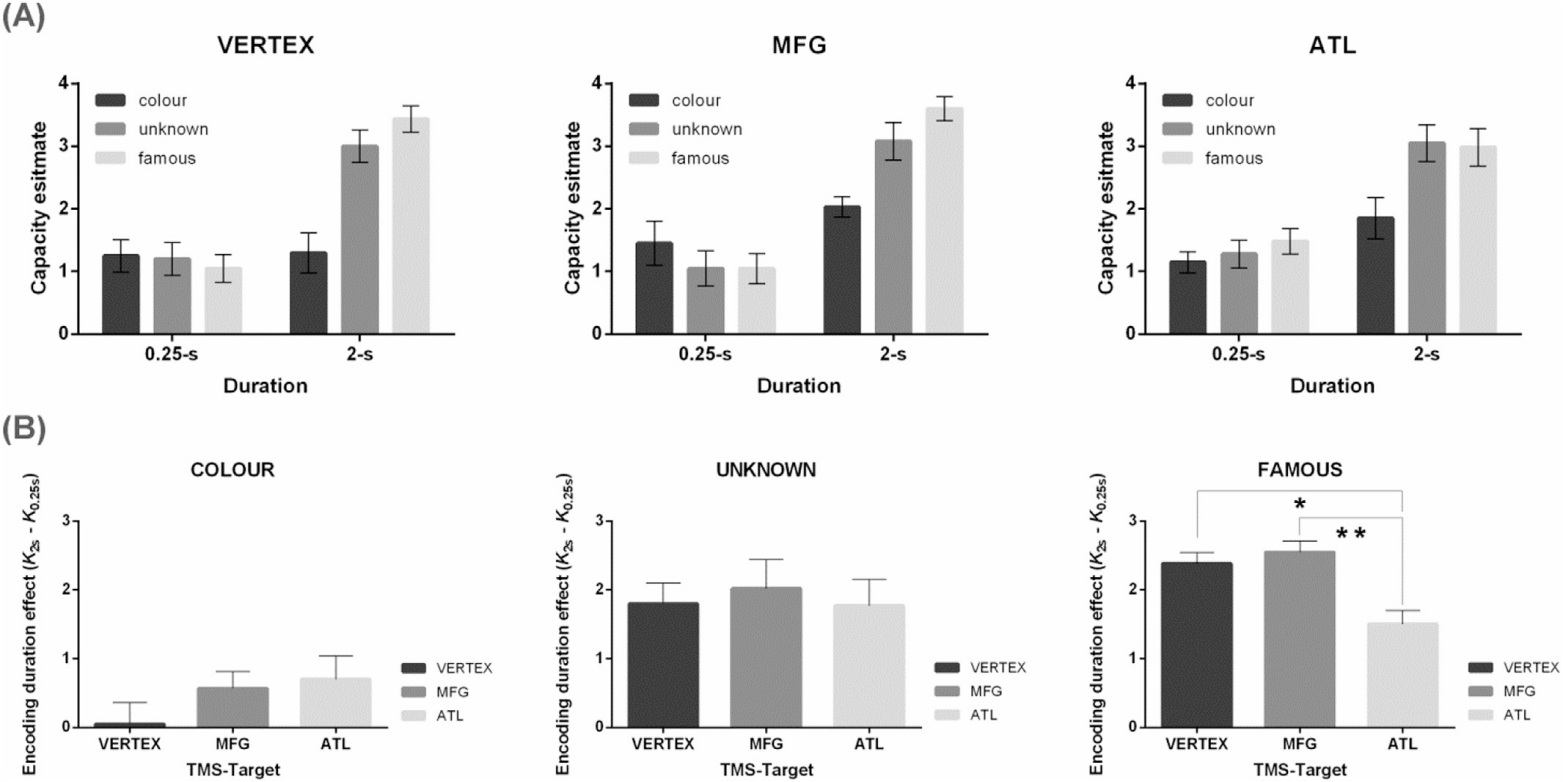
(A) Capacity estimates, Cowan's *K*, displayed as a function of the types of stimuli by encoding durations by TMS sites. (B) The interaction between encoding durations and TMS sites was only significant in the famous face condition. Asterisks represent significant pair-wise comparisons; * represents 0.05 > *p* > .01; ** represents 0.01 > *p* > .001.

Participants performed well on the digit task, achieving mean accuracy of 90% (SD: 3%). The capacity estimates (Cowan's *K*) of every condition are displayed in Fig. 4A. For different stimuli, we examined how theta-burst stimulation modulates WM capacity as a function of different brain regions × different encoding time. In the condition of coloured squares (see Fig. 4B left), we found that WM capacity for coloured squares did not seem to significantly benefit from longer encoding time (*F*_(1,11)_ = 3.72, *p* = .08, *η*_*p*_^2^ = 0.25), despite a slight trend consistent with Experiment 1 and previous literature. In addition, TMS did not modulate WM for colours (*F*_(2,22)_ = 1.18, *p* = .33, *η*_*p*_^2^ = 0.1), nor did the interaction of TMS × Encoding Duration (*F*_(2,22)_ < 1, *p* = .43). In the condition of unknown faces (see Fig. 4B middle), we found that longer encoding time is beneficial to remembering unknown faces (*F*_(1,11)_ = 77.34, *p* < .001, *η*_*p*_^2^ = 0.87), replicating the significant facilitatory effect we had observed in Experiment 1. Brain stimulation, however, did not affect WM for unknown faces, nor did the interaction of TMS × Encoding Duration (both *F*_(2,22)_ < 1, *p*s > 0.91).

Strikingly different patterns were seen in the results of famous-face condition: Longer encoding time led to significantly greater WM capacity (*F*_(1,11)_ = 129.67, *p* < .001, *η*_*p*_^2^ = 0.92). Critical to our aim to examine the modulatory impact of cTBS, we found a significant interaction of TMS × Encoding Duration (*F*_(2,22)_ = 6.62, *p* = .006, *η*_*p*_^2^ = 0.38). Based on the significant interaction, we carried out further *post-hoc* comparisons: While the effect of Encoding Duration was significant in all of the three TMS conditions (all *p*s < 0.001), the magnitude of this effect was modulated by applying cTBS to different sites. Right-ATL stimulation significantly attenuated the facilitation of longer encoding time, compared to right-MFG stimulation (*t*_(11)_ = 3.15, *p* = .009, Cohen's *d* = 0.909) and vertex stimulation (*t*_(11)_ = 2.61, *p* = .024, Cohen's *d* = 0.753). There was no difference between the MFG and vertex condition (*p* > .5). As illustrated in the rightmost part of Fig. 4B, while prolonging the time to encode led to an overall gain in WM capacity, the additional information accumulated over the 1.75 s (measured as the difference *K*_2.0_ – *K*_0.25_) significantly declined by a margin of 39% after ATL stimulation relative to MFG stimulation (tested against zero/no reduction: *t*_(11)_ = 3.20, *p* = .008, Cohen's *d* = 0.925) and by 34% relative to vertex stimulation (tested against zero: *t*_(11)_ = 2.24, *p* = .04, Cohen's *d* = 0.648).

To ascertain whether a lack of statistical power underlay the selective perturbation of ATL stimulation (*vs.* the absence of MFG stimulation effect), we performed power calculations. The comparisons against the control site vertex provides a critical baseline to assess this possibility. Relative to vertex stimulation, ATL stimulation significantly mitigated the semantic facilitation impact (*t*_(11)_ = 2.61, *p* = .024); by contrast, MFG stimulation had no influence (*t*_(11)_ = −0.695, *p* = .501). To gauge the size of power, we calculated for the ‘vertex *vs*. ATL’ contrast that revealed a significant TMS effect – under its effect size (*D*=0.753), Type-I error probability (*α* = 0.05), sample size (*n* = 12) – the power (1–*β*) is 0.79. According to a comprehensive meta-analysis that surveyed 26,481 *t*-test records from 3801 recent papers from the neuroscience and experimental psychology literature, the median effect size and power are *D* = 0.65 and 1–*β* = 0.7, respectively (Szucs and Ioannidis, 2017). Relative to the meta-analysis results, our ATL stimulation resulted in a substantially larger effect size than the median of majority of studies, and our power was also evidently larger than the bulk of literature. This means that we did have sufficient statistical power to detect any effect, if it truly exists. Critically, the effect size of the “vertex vs. MTG” contrast (*D* = 0.201) was considerably weaker than the *D* of ATL stimulation and would be deemed as a small effect by traditional criterion (Szucs and Ioannidis, 2017), implying a low possibility for this effect to genuinely exist. Furthermore, under our sufficient power with all other parameters being identical, a huge sample of *n* = 151 is estimated to be necessary to detect the ‘vertex vs. MTG’ effect if we assumed it exists in reality. Together, these additional analyses ascertain the trustworthiness of ATL stimulation disrupting WM storage and guard against a lack of statistical power being the reason of the selective effect.

In addition to *K*, we calculated accuracy (proportion of correct responses) and tested whether its slope (accuracy as a function of Encoding Duration) was modulated by TMS. The results are entirely consistent with *K*: For both coloured squares and unknown faces, the slopes (accuracy by time) of the three TMS conditions did not differ (colour: *F*_(2,22)_ = 0.94, *p* = .41, *η*_*p*_^2^ = 0.08; unknown: *F*_(2,22)_ = 0.09, *p* = .91, *η*_*p*_^2^ = 0.01), suggesting that both were immune to TMS modulation. Critically, for famous faces, TMS significantly modulated the slope of accuracy by Encoding Duration (*F*_(2,22)_ = 6.22, *p* = .007, *η*_*p*_^2^ = 0.36); *post-hoc* tests revealed that ATL stimulation attenuated the steepness of slope (i.e., the facilitatory impact of semantic knowledge dwindled following disruption to the ATL), relative to vertex and MFG stimulation (both *p*s < 0.05), while no difference was found between vertex and MFG stimulation (*p* = .49). Together, multiple analyses coherently suggest that ATL stimulation genuinely produced a selective and causative influence to hinder the rate of increment for famous faces in the WM buffer.

## Discussion

In this study, we present three principal findings about the functional properties of WM and its causative neural substrate. First, we found that WM capacity continues to increase for human face stimuli over a timescale of 2 s, whereas its capacity for coloured shades plateaus within 0.25-s encoding time.^1^ Second, with the length of encoding time and visual distinctiveness being matched, the WM system was able to maintain more famous faces than unknown faces. Third, using theta-burst stimulation to investigate the neural locus that engenders the advantage of famous faces, we showed that disrupting the right ATL potently weakened the facilitatory effect by a significant margin of ∼35%. With careful control over low-level pictorial features, we ensured that the superiority of famous faces over unknown faces cannot be attributed to any perceptual explanation. Stimulation to the right ATL selectively disrupted the WM processing of famous faces while *not* affecting unknown faces and colours, showing that this brain area is specifically engaged when WM content carries semantic information about individuals. Moreover, neither right-MFG nor vertex stimulation affected WM capacity for any type of materials, ruling out non-specific impacts of cortical stimulation that might alter arousal level or cause distraction. Our data replicated the WM advantage for faces reported previously (Curby and Gauthier, 2007), and concur with prior findings that WM expands due to abstract concepts regarding the regularities of object position (greater WM for normal than inverted objects: Kaiser et al., 2014, Kaiser et al., 2015). Furthermore, we demonstrated the striking advantage that semantic knowledge confers to famous faces and pinpointed the causative neural origin underlying this advantage. We note that the overall WM capacity we observed is lower than the estimates obtained by Brady et al. (2016). We posit that the discrepancy is driven by difficulty. Specifically, whereas we used stimuli of similar colours within an array (shades of different blue, green, or skin colours), Brady et al. used stimuli of conspicuously distinct colours that facilitate detection as a change of stimulus would be highly noticeable; their objects were distinctly shaped, which might also help encoding or differentiating. Despite differences in overall capacity, our results are consistent with the pattern found in Brady et al.’s data that WM storage keeps growing with encoding time, selectively for meaningful stimuli.

### Advancement from correlation to causation

Brady et al. (2016) showed that the reinforced WM capacity for real-world objects is associated with greater amplitude of the contralateral delay activity (CDA). The CDA has been shown to correlate with how much information is kept in the WM buffer and shrinks in amplitude when the information has been consolidated into long-term episodic memory (for review, see Fukuda et al., 2010), with its major neural origin estimated to the parietal lobules by source localisation analysis (Becke et al., 2015). Thus, the CDA evidence enabled Brady et al. to conclude that the boost to WM capacity cannot be solely explained by episodic memory but rather it reflects genuine improvement of WM *per se*. However, while their data compellingly show expanded WM for objects, it is limited by EEG's correlative nature and imprecise spatial resolution. In addition, it is unclear whether the greater CDA amplitude detected over parietal electrodes results from top-down modulatory signals from the higher-level regions of executive control or semantic knowledge, such as the PFC or ATL. Advancing beyond such correlative inferences, our TMS findings enable us to establish the causative functional linkage between the ATL and WM capacity – the right ATL, a region known for its role in representing semantic knowledge including person identity (e.g., Binney et al., 2016, Lambon Ralph et al., 2017, Yang et al., 2016), causally mediate WM expansion for faces that are highly recognisable and carry semantic attributes. By contrast, while the MFG and its neighbouring regions in the PFC show enhanced connectivity with occipitotemporal face areas during WM, it does not causally contribute to the facilitatory effect of faces on WM.

### The ATL supports high-dimensional representations that benefits WM

We speculate that the role of the ATL is *not* to directly enhance encoding, retention, or retrieval of WM *per se*; instead, it supports semantic representations (person knowledge) that affix each face to a unique individual identity and other person-related attributes. Such semantic representations might help individuate each stimulus in working memory, making their neural coding more separable, rather than partially overlapped, in an abstract representational space. Thus, while the ATL might not directly underpin WM, it supports semantic knowledge that boosts WM. Our view is consistent with the multi-dimensional hypothesis by Wyble et al. (2016). In their hypothesis, conceptually meaningful information is represented with higher dimensionality than meaningless geometric shapes. In the example of coloured squares, the neural coding for such stimuli would rely on only two perceptual-level dimensions – colour and location, and consequently different items in a visual array have less distinct patterns of coding (hence, are more confusable). By contrast, meaningful stimuli, such as faces or objects, enjoy the advantage of having additional dimensions that allow more distinct neural coding. In particular, our comparisons of famous and unknown faces further demonstrate the striking impact of such additional semantic-level dimension while the perceptual properties of two sets of stimuli has been rigorously matched. Our TMS data provide crucial evidence bridging Wyble et al.’s view of multi-dimensional coding with a prominent theory about the neurocomputational basis of semantics (Lambon Ralph, 2014, Lambon Ralph et al., 2017). Specifically, we showed that disrupting the right ATL selectively mitigates the impact of famous faces. This is echoed with the deficits of patients with semantic dementia (SD), which results from degenerative atrophy of the ATLs, bilaterally. Atrophy of this region is associated with progressive degradation of multimodal semantic knowledge leading to erosion of the boundary between concepts (Lambon Ralph et al., 2010). SD patients fail to distinguish between closely-related concepts, such as foxes *vs*. wolves. Whilst naming items they resort to generic, superordinate-level terms (e.g., ‘animal’) to refer to a broad collection of related concepts, due to blurred division between specific entities (or a drastic reduction in the dimensions available to represent different concepts). As a part of their semantic deficits, SD patients lose the ability to differentiate between the faces of famous individuals while preserving normal ability to perceive faces, suggesting a loss of person knowledge driven by ATL atrophy (Simons et al., 2001, Snowden et al., 2004). These neuropsychological patient data and other convergent neuroimaging data (see Lambon Ralph et al., 2017) fit with the present TMS finding, lending support to our interpretation that TMS causes a temporary and moderate form of virtual lesion, mimicking the loss of face knowledge in SD patients.

### The roles of PFC in working memory

The results of PFC stimulation ensure the selectivity of ATL stimulation in perturbing WM advantage for famous faces. Applying cTBS to this control site resembles ATL stimulation in tactile sensation but this region does not represent WM content and semantics *per se*. While it provides a suited control context underscoring the selectivity of ATL, it is worth discussing why WM performance was unaffected following PFC stimulation. We posit two plausible explanations. One possibility is the variability in the functional localisation of PFC – relative to ATL's anatomy, locating a PFC ‘hotspot’ for TMS is more difficult because the mapping between WM functions and PFC-sites may be more variable across individuals. The other more interesting possibility is the choice of tasks: As discussed earlier, the PFC shows persistent activity during the retention period of WM (for review, see Sreenivasan et al., 2014), and it is interpreted as producing modulatory signals that augment or dampen other regions where WM content is stored. Interestingly, such findings are usually based on *n*-back paradigms that require continual updating (e.g., sustaining new items in the WM buffer while discarding antecedent items), and the PFC involvement has been interpreted as evidence of executive control during WM (for review, see Lara and Wallis, 2015, Postle, 2016). Critically, however, the extent of PFC involvement monotonically varies with the type of WM operation: Meta-analysis has shown that tasks involving manipulation (updating and reordering of WM content, such as *n*-back) are more likely to elicit PFC activity, compared to a task that does not require updating, such as the paradigm we used in the present study (Wager and Smith, 2003). Patient data also offer hints that the PFC's involvement in WM processing depends on tasks – individuals with large PFC lesion showed little impairment on tasks that do not require active updating, such as digit span or delayed recognition (e.g., D'Esposito and Postle, 1999, Müller and Knight, 2006, Müller et al., 2002). Thus, while there is evidence of enhanced connectivity between the PFC and occipitotemporal face regions during WM, it might rely upon the specific task requirement of active updating and deliberate omission (e.g., keeping newly registered faces while ignoring previous faces and scenes; see Bollinger et al., 2010). Taken together, this large literature offers an intriguing explanation: A task that does not require constantly updating WM might be relatively resistant to PFC stimulation. By contrast, our paradigm is sensitive enough to capture the effect of ATL stimulation on disrupting semantic facilitation.

### Convergent evidence for the influence of semantic knowledge on verbal WM

There is a parallel, established literature on the impact of semantic knowledge on verbal WM (Jefferies et al., 2006c; Jefferies et al., 2006a, Jefferies et al., 2006b, Jefferies et al., 2008, Jefferies et al., 2005, Patterson et al., 1994). For example, when probed with a same-different matching task, healthy participants showed better recall for real words than pseudo-words, and for concrete than abstract words, suggesting the influence of lexical-semantic knowledge that helps to enhance immediate serial recall. Further, mirroring our TMS data, task performance of patients with semantic dementia (arising from ATL atrophy) shows a significant reduction of the semantic effect on their verbal WM. By considering the literature on verbal and visual working memory together, the separate lines of inquiry suggest that there is a domain-general role of ATL in supporting WM, irrespective of whether the content is verbal or visual.

## Conclusion

By disturbing neural processing of targeted cortical regions using TMS, we showed that virtually lesion the right ATL causally reduces the benefits to WM brought about by famous faces whiling leaving the effect of unknown faces intact. The specificity of the ATL in mediating these effects was supported by the fact that MFG and vertex stimulation did not modulate WM performance. These data suggest that the ATL, a neural structure that has been neglected as a possible node of the WM network by the field of vision sciences, contributes to buttressing WM storage for meaningful information, via the mediation of semantic knowledge. Together, our findings point at a fruitful future direction to investigate the neural basis that underpins the WM processing of socially meaningful information.

## Conflicts of interest

The authors declare no competing financial interests.

## Footnotes

1 It is crucial to note how the disparity of the encoding process for colours *vs*. faces might interact with encoding duration and face recognition. Specifically, the appearance of colours is potentially influenced by the simultaneous presence of other hues in the display. Thus, perceived colour would alter depending on the particular spatial arrangement of the hues (i.e., a context effect). By contrast, faces might be less affected by such contextual effects because face identity can be encoded independently of the identities of other faces. This disparity in encoding for the two stimulus types might partially explain the differences in WM capacity – when a colour patch is shown in isolation to probe WM, it lacks other colours that accompanied it in the original encoding context. This could lead to lower WM performance in comparison to that of faces (which do not rely on the context formed with co-presented stimuli during encoding). However, it is important to stress that while the encoding disparity could potentially contribute to different WM capacities when encoding duration is long (2 s), it is less likely to have an influence when duration is brief (0.25 s). This pattern is reliably observed in both Experiments 1 and 2; when the presentation is brief, WM capacity (*K*) of colours is comparable to that of faces despite the encoding process presumably differing between colours and faces. The difference in *K* only emerges when encoding time is long enough for face recognition (and knowledge about person identity by extension) to be completed and facilitate encoding, thus subsequently enlarging WM storage.
